# Acetylation and phosphorylation control both local and global stability of the chloroplast F_1_ ATP synthase

**DOI:** 10.1038/srep44068

**Published:** 2017-03-09

**Authors:** Carla Schmidt, Victoria Beilsten-Edmands, Shabaz Mohammed, Carol V. Robinson

**Affiliations:** 1 Department of Chemistry, University of Oxford, Oxford, United Kingdom; 2 Department of Biochemistry, University of Oxford, Oxford, United Kingdom

## Abstract

ATP synthases (ATPases) are enzymes that produce ATP and control the pH in the cell or cellular compartments. While highly conserved over different species, ATPases are structurally well-characterised but the existence and functional significance of many post-translational modifications (PTMs) is not well understood. We combined a range of mass spectrometric techniques to unravel the location and extent of PTMs in the chloroplast ATP synthase (cATPase) purified from spinach leaves. We identified multiple phosphorylation and acetylation sites and found that both modifications stabilise binding of ε and δ subunits. Comparing cross-linking of naturally modified cATPase with the *in vitro* deacetylated enzyme revealed a major conformational change in the ε subunit in accord with extended and folded forms of the subunit. Locating modified residues within the catalytic head we found that phosphorylated and acetylated residues are primarily on α/β and β/α interfaces respectively. By aligning along different interfaces the higher abundance acetylated residues are proximal to the regulatory sites while the lower abundance phosphorylation sites are more densely populated at the catalytic sites. We propose that modifications in the catalytic head, together with the conformational change in subunit ε, work in synergy to fine-tune the enzyme during adverse conditions.

Rotary F-, V- and A-type ATP synthases are enzymes that produce or hydrolyse ATP and thereby control the pH in the cell^1^. They are embedded in compartmental or plasma membranes of eukaryotes, bacteria or archaea and share a common architecture comprising a soluble F_1_/V_1_/A_1_ and a membrane-bound F_O_/V_O_/A_O_ domain^2^. Recent breakthroughs in cryo-electron microscopy delivered for the first time high-resolution structures of intact ATPases^3^, their conformational states^4^ and combined with cryo-electron tomography novel insights into dimer formation in natural membranes^5^,^6^.

Much less well studied than the eukaryotic and bacterial enzymes is the cATPase, located in the thylakoid membrane of plant chloroplasts. cATPase utilises a proton gradient established during photosynthesis for ATP production. The F_1_ soluble ‘head’ of cATPase contains the α, β, γ, δ, and ε subunits. F_O_ is composed of the membrane ring (III_14_) and subunit IV. The catalytic centre of the cATPase is represented by the α/β-‘head’ with catalytic (β/α) and regulatory (α/β) nucleotide binding sites at the interfaces. The rotor of the enzyme comprises the membrane ring (III_14_) together with γ and ε subunits. The “head” and the rotor are linked by a peripheral stator stalk containing subunits δ, I, II and IV^7^.

To date there are relatively few reports of PTMs and their functional role in ATP synthases. However PTMs are involved in a variety of cellular functions and have emerged as being essential for eukaryotic life^8^. Understanding the interplay between various PTMs and the ways in which they regulate cellular function is therefore of paramount importance. The advent of mass spectrometry-based proteomics allows the identification^9^ and quantification^10^ of PTMs and delivers essential information on their functional role during the cell cycle. Of the numerous modifications that can occur phosphorylation and acetylation are the most extensively studied in large-scale proteomics experiments (for reviews see refs 11, 12, 13). Cross-talk between these important modifications has also been proposed (e.g. refs 14, 15, 16) but the impact of PTMs at the structural level can be difficult to define.

Turning to the ATP synthases the most prominent PTM reported to date is trimethylation of Lysine-43 in the membrane ring subunit, which was found to be complete and conserved across several vertebrate species^17^,^18^. This particular PTM is located in a loop between the two α-helices of the ring subunit and proposed to be involved in cardiolipin binding in the mitochondrial membrane^19^. Recent studies have also linked PTM status with stress, particularly in bovine mitochondrial ATP synthase. Both nutrient and exercise induced stress were linked to increased deacetylation of the ATPase via SIRT3, with a particular impact on OSCP, the homologue of subunit δ in cATPase^20^.

cATPase has been the target of proteomic investigations which have focussed primarily on PTM regulation in the absence and presence of light. For example subunit β was identified as a target of Casein Kinase II in the dark period^21^,^22^. Previously we investigated the role of phosphorylation in the intact cATPase. By comparing populations of naturally modified and dephosphorylated enzymes we found that phosphorylation of the cATPase has an effect on complex stability and nucleotide binding in the catalytic interface. Using comparative cross-linking^23^ we also identified conformational changes in the ε subunit which acts as a brake to prevent free rotation of the head, and in the C-terminal regions of the α/β interface, which controls access to nucleotide binding sites^24^.

Here we extend our knowledge of the role of PTMs in ATP synthases. We identify novel phosphorylation and acetylation sites in the chloroplastic enzyme and determine their occupancy levels. Comparing mass spectra of the naturally modified and deacetylated cATPase exposes both similarities and differences between the two modifications. Specifically, we found that the deacetylated cATPase is less stable and that nucleotide occupancy is decreased after partial deacetylation. Importantly the location of the highest abundance modification sites in the structure of the cATPase suggests that acetylation and phosphorylation control different interfaces in the α/β catalytic core as well as differentially modulate interactions with the δ and ε subunits.

## Results

### Identifying PTMs in the cATPase

**Figure 1:**
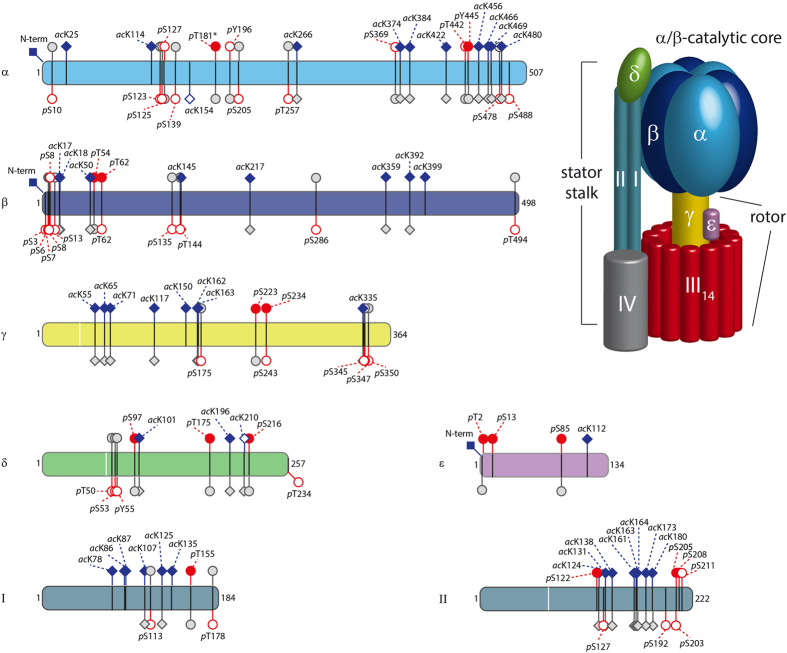
PTMs identified in cATPase purified from spinach chloroplasts. Protein subunits are represented as bars. N-terminal transit peptides are indicated by a white line. PTMs identified in cATPase (above bars) and in *Arabidopsis thaliana* (below bars) are shown. Acetylation sites (blue squares) and phosphorylation (red circles) sites are shown. PTMs identified in this study are filled. PTMs with conserved residues are shown in grey. Note that amino acid sequences of both species were aligned and residue numbers are shifted according to the alignment.

We purified chloroplasts from spinach leaves and extracted cATPase using a protocol described previously^24^,^25^. Next, we digested cATPase with trypsin to obtain peptides for LC-MS/MS analysis. We employed a high-resolution, high-speed mass spectrometer to allow for PTM identification without additional enrichment steps (Methods). After database searching and inspection of the MS/MS spectra we identified 63 modified sites. Of these, only seven acetylation and two phosphorylation sites had been reported previously^24^ (Fig. 1 and Supplementary Table S1). In detail we identified 46 acetylated lysine residues, the acetylated N-termini of the α, β and ε subunits, and 14 phosphorylated sites, on one tyrosine, six threonine and seven serine residues. In total, eight of the nine protein subunits were modified; only membrane embedded subunit III was found to be unmodified in our experimental conditions.

Comparing PTMs in the ATPases from spinach and mouse-ear cress (*Arabidopsis thaliana*) chloroplasts we observed a high level of conservation. We found co-location of modifications in conserved regions together with similar sequence stretches without modification (Fig. 1). Of particular note are the clusters of modifications in both species at the N and C termini of the β and α subunits respectively, as well as the C-terminus of subunit II. These extensive modifications in the soluble head of the cATPases from both spinach and *Arabidopsis thaliana* imply critical roles for PTMs in influencing the stability of the F_1_ head.

### Acetylation is required for stable binding of folded subunit ε

To determine the effects of acetylation on subunit interactions we compared an aliquot of cATPase after incubation in buffer containing a deacetylase with an aliquot of the cATPase in its untreated form, i.e. incubated in buffer for the same time period. For deacetylation we chose SIRT3, a member of the mammalian Sirtuin family. This particular deacetylase is a mitochondrial enzyme and has been shown to deacetylate mitochondrial ATP synthase in human^26^ and in bovine^20^ as well as proteins in *Arabidopsis thaliana*^27^.

**Figure 2:**
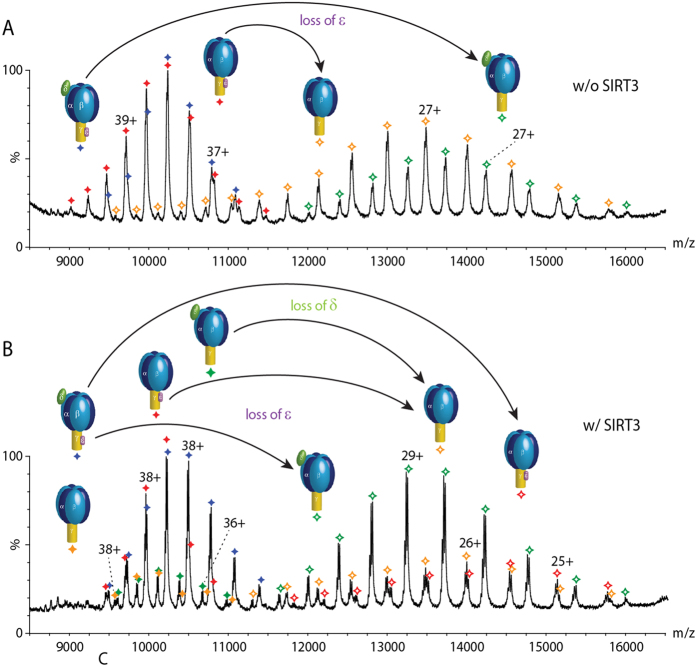
Acetylation is important for binding of δ and ε subunits as well as overall complex stability. (**A**) A typical mass spectrum of the naturally modified cATPase. The intact F1 and the complex that had lost the δ subunit are present in solution. Dissociation products are generated in the gas-phase due to loss of ε. (**B**) After treatment with a deacetylase (SIRT3) two more complexes that have lost the ε subunit are present in solution. Gas-phase dissociation products are generated by loss of ε and δ subunits. Intensities of dissociation products are increased.

**Figure 3:**
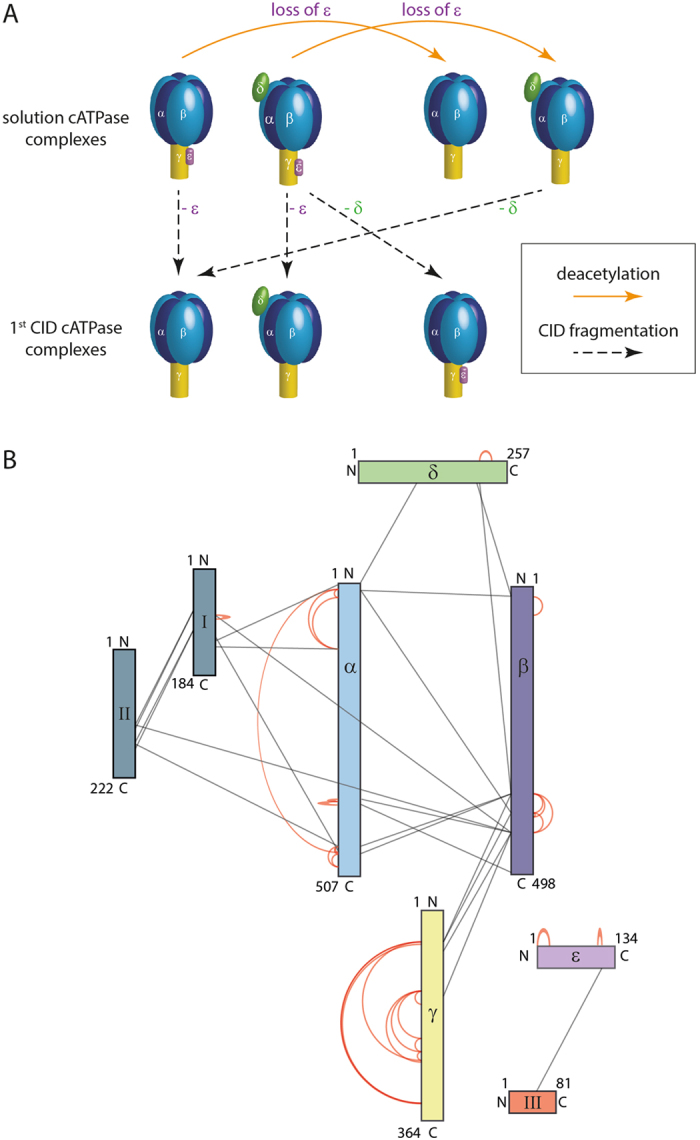
Dissociation pathways and interaction network of acetylated/deacetylated cATPase. (**A**) After deacetylation loss of subunit ε in solution is observed. Gas phase CID products reveal loss of subunits ε and δ. (**B**) Interactions identified in cATPase are indicated by black lines. Intra-molecular interactions are denoted by red lines. Protein interactions were obtained from comparative cross-linking experiments.

We first acquired a spectrum of the untreated cATPase using a mass spectrometer modified for transmission of large protein assemblies^28^. Two species were present in solution under the conditions of this experiment, the intact F_1_ complex as well as a sub-population in which the δ subunit has dissociated in solution (Fig. 2A). We also observed gas phase dissociation products (m/z 12 000–16 000) which we assigned to loss of the ε subunit from both parent complexes (Fig. 2A). Comparing this spectrum with the one recorded after incubation with SIRT3 we find that the intensity of the peaks assigned to gas phase dissociation products has increased and is now of equal intensity to the parent complexes (Fig. 2B). Considering the assignment of the complexes formed after treatment with SIRT3 in solution, spectra are broadly similar to those before treatment, losses of δ and ε subunits are apparent, as before (Fig. 2B). Two additional complexes, both generated by dissociation of the ε subunit in solution following treatment with SIRT3 are also present, albeit at lower intensities. In the gas phase dissociation region however the complex in which ε is lost becomes the predominant product. A second species, in which δ also dissociates is observed (Fig. 2B). Together these results imply facile dissociation of subunit ε and, to a lesser extent, subunit δ following deacetylation (Fig. 3A).

**Figure 4:**
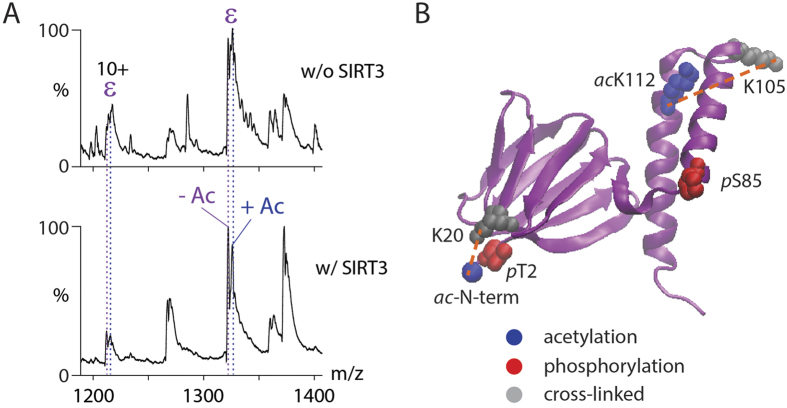
cATPase-ε is acetylated. (**A**) Two populations of ε were observed in low m/z mass spectra: the acetylated (+Ac) and deacetylated (−Ac) ε subunit. Before SIRT3 deacetylase treatment approximately equal abundances were observed for the two populations (upper panel). After incubation with SIRT3, intensities for the unmodified population increased (lower panel). **(B)** A homology model of ε is shown^23^. Phosphorylated (red) and acetylated (blue) residues are shown (space fillings). Lysine residues that were cross-linked are shown (grey) and cross-links are highlighted (dotted lines).

Expansion of the low m/z region of the 9+ charge state of the ε subunit (m/z ~1340) before and after deacetylation reveals peak splitting which corresponds to acetylated and deacetylated forms of the ε subunit (Fig. 4A). After incubation with SIRT3, the peak intensities of the lower mass species, corresponding to the unmodified ε subunit, increase showing that the extent of acetylation is decreased. Although the deacetylase reaction does not go to completion, the resolution of the charge state peaks is enhanced since overlap of multiple acetylated forms is reduced following this deacetylase reaction.

To investigate the importance of acetylation for stable protein interactions we employed a comparative cross-linking strategy described previously^23^. In this procedure the naturally modified (untreated) ATP synthase was incubated with non-deuterated bis(sulfosuccinimidyl)suberate (BS3-d0) while the deacetylated ATP synthase was incubated with deuterated BS3 (BS3-d4). Following chemical cross-linking the two populations were combined and processed together (see Methods). After database searching and manual validation of mass spectra, we were able to quantify 54 protein interactions; 25 of these represent inter-subunit interactions (Fig. 3B and Supplementary Table S2). Calculating ratios of intensities (d0/d4) for the protein interactions before and after deacetylation revealed that the majority of cross-linked interactions are not affected by the removal of acetylation sites from the complexes. Other interactions showed slight changes in intensities (<0.5 d0/d4 > 2.0) (Supplementary Table S2).

Many of these cross-linked peptides were found to be acetylated. Since acetylated amino groups will not be available for cross-linking we anticipate an increase in cross-linking following deacetylation if no conformational changes take place. A significant decrease in intensity was observed however for two intra-protein interactions within the ε subunit itself following deacetylation. The cross-link located in the hinge region of the anti-parallel double-α-helix decreased by a factor of >50 (K105:K112) while the N-terminus and the adjacent β-domain (N-term:K20) reduced by a factor of seven (Fig. 4B). This decrease in cross-linking is consistent with two possible scenarios (i) solution phase dissociation and unfolding of subunit ε or (ii) conformational change of subunit ε while retained by the complex. We reasoned that if the ε subunit dissociates in solution it will be apparent ~10,000 m/z, without gas phase dissociation; conversely if the minus ε population increases significantly following CID, this is consistent with an elongated form of subunit ε dissociating more readily in the gas phase. It is established that extended subunits dissociate more readily than compact ones during CID of protein complexes^29^,^30^. We find that losses of the ε subunit are more prevalent in both phases following deacetylation. However, the gas phase product, in which the δ subunit is retained is predominant, compared with loss of the ε subunit following deacetylation (Figs 2B and 3A). This facile dissociation is consistent with the dramatic reduction in cross-linking observed for the ε subunit. Since conformational change is an important switch between the active (compact) and inactive (extended) states^31^,^32^,^33^,^34^, this extension of the hinge region of subunit ε is consistent with reduced activity in response to deacetylation.

### Effects of deacetylation and dephosphorylation on nucleotide binding

**Figure 5:**
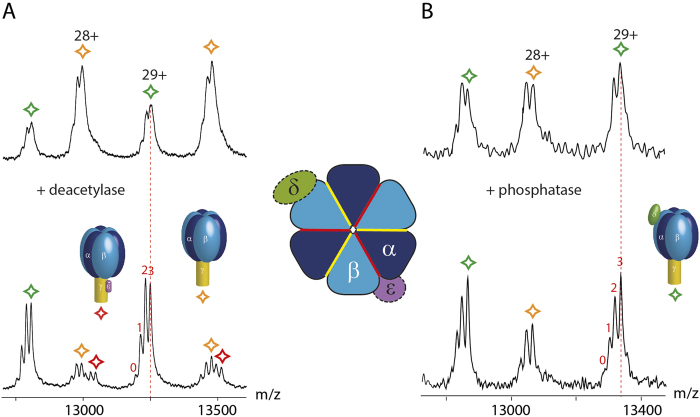
Nucleotide binding of cATPase is regulated by PTMs. The untreated cATPase shows two populations with two or three nucleotides bound (upper panels). After deacetylation (**A**) and dephosphorylation (**B**) additional populations corresponding to cATPases with zero and one nucleotide bound are observed. Interestingly ε is retained following deacetylation, but not phosphorylation, consistent with cross-linking data and its extended form. Intensities of the peaks corresponding to two nucleotides bound increased, specifically in the case of the deacetylated complex. Panel (B) was modified from^24^.

Expansion of the peaks of the intact F_1_ head and subcomplexes reveals populations that differ by the mass of a single nucleotide (Fig. 5). Within the context of these large complexes it is not possible to distinguish ATP from ADP, or a combination of both. It is clear however that there is a maximum of three bound nucleotides (ADP/ATP) in the F_1_ head (Fig. 5). The exact binding sites of these nucleotides is unknown and cannot be compared directly with x-ray data where excess nucleotides are added during crystallisation^35^. The bound nucleotides observed here survive our isolation protocol and are partially resolved in the mass spectra of the wild-type complex. To investigate the effects of deacetylation on the stability of these bound nucleotides we incubated cATPase with SIRT3. The additional complex observed after activation in the gas phase through retention of subunit ε is seen clearly here following deacetylation (Fig. 5A, lower panel red) consistent with data above. Considering the effects on nucleotide binding we find that up to three nucleotides remain bound following destabilisation of the δ and ε subunit after incubation with deacetylase. Interestingly new peaks corresponding to binding of one and zero nucleotides are also observed, as shown previously following dephosphorylation^24^. Comparing these results with dephosphorylation we find that the loss of the δ and ε subunits occurs to a lesser extent following deacetylation while the two-nucleotide bound state predominates. Formation of states with less than two nucleotides is also observed, in line with the effects seen following dephosphorylation, and likely arising from destabilisation of the F_1_ complex.

### Combining the effects of dephosphorylation and deacetylation

These conclusions raise the question of how these complexes are affected when levels of both acetylation and phosphorylation are reduced simultaneously. We compared cATPase incubated without SIRT3 and CIP (calf intestinal phosphatase) with cATPase subjected to incubation in the presence of SIRT3 and CIP. Mass spectra, recorded under the same conditions, are complicated since the stability of the deacetylated/dephosphorylated complex was compromised (Figure S1).

To quantify this combined effect we applied comparative cross-linking to investigate changes in protein interactions after simultaneous deacetylation and dephosphorylation. As above, naturally modified ATP synthase and the unmodified ATP synthase were cross-linked with BS3-d0 and BS3-d4, respectively. Most of the cross-linked peptides contain one, or even multiple modifications, hampering this experiment. Nonetheless, we were able to quantify 19 protein interactions including eight inter-protein interactions (Supplementary Table S3). If we compare the results from our deacetylation cross-linking experiments with those in which we have carried out both deacetylation and dephosphorylation we find that incubating with the deacetylase alone causes only moderate loss of intra-protein interactions, an effect that is primarily observed for subunit ε. By contrast simultaneous deacetylation and dephosporylation induces considerable dissociation with all comparative cross-links changing significantly (the majority >2 and <8). The decrease in intra-protein interactions identified here is induced by deacetylation while on a wider scale dephosphorylation destabilises a greater range of subunit interfaces.

These findings suggest a role for phosphorylation in stabilising the protein complex globally and for acetylation in stabilising protein subunit interactions and conformational changes locally, specifically in subunits ε and δ. Furthermore, we can conclude that the effects of removing both modifications simultaneously appear to be more significant than the sum of the effects observed when modifications are removed separately.

### Location of the PTMs within different interfaces

**Figure 6:**
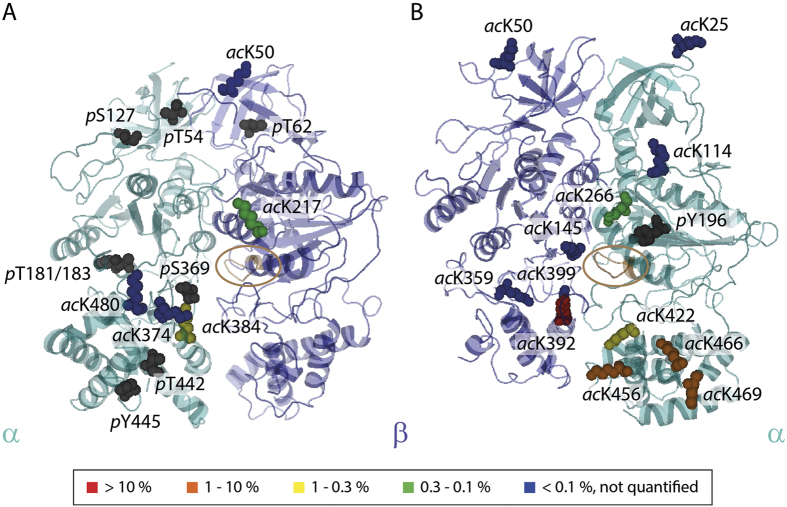
Protein interfaces in the catalytic center of the cATPase are regulated by PTMs. Phosphorylation and acetylation sites are shown (space fillings) on the crystal structure of the cATPase α/β-head (PDB ID 1FX0). Acetylation sites are coloured according to their abundance (see heatmap). (**A**) The α/β-interface of the catalytic head is dominated by phosphorylation sites (grey). Acetylation sites are low abundant. (**B**) The majority of acetylation sites was identified in the β/α-interface. Abundant sites are located in the C-terminal domain important for nucleotide binding.

We next located the modified residues in the available crystal structures and homology models^24^. Most of the identified sites are located at protein interfaces in accord with their importance for complex stability (Supplementary Figure S2). Of the 63 modified sites, 24 could be projected onto the available crystal structure of the α/β-head (Fig. 6). The remaining 39 are located within unstructured regions not included in X-ray structures or in subunits without high-resolution structures. With one exception (*p*Y196), all phosphorylation sites are located in the α/β-interface harbouring the catalytic nucleotide binding site (Fig. 6A and B). By contrast acetylation sites are more widely spread throughout the F_1_ head with the majority being identified in the β/α regulatory interface. Interestingly, these sites are mostly located in the middle and C-terminal domains of α and β subunits (Fig. 6) suggesting that they may regulate access to nucleotide biding sites. Comparing acetylation and phosphorylation sites identified in spinach leaves and in *Arabidopsis thaliana* reveals that they co-localise in the N-terminal, middle- and C-terminal domains (Fig. 1) and that most residues that are modified are conserved within both species.

An important aspect when studying PTMs is the abundance of the modified sites. Knowing the absolute occupancies (i.e. the intensity ratio of phosphorylated or acetylated peptide-to-unmodified peptide) allows conclusions to be drawn on the impact of individual sites in the functionally active ensemble. We determined the occupancy of modified sites following an intensity-based approach (Methods)^36^,^37^. Of the 63 modified sites identified here, we were able to quantify the occupancy of 41 sites absolutely, including both PTMs phosphorylation and acetylation (Supplementary Table S4). On average 1.91% of the acetylated sites are modified with some of the sites being heavily modified (>3%). Phosphorylation instead is of very low abundance in our experiments with only 0.21% occupancy of the phosphorylated sites (Fig. 5A and B and Supplementary Table S4). Overall, we conclude that acetylation is not only more prevalent than phosphorylation (~3 fold) but is also more abundant (~10 fold) than phosphorylation.

The most abundant acetylation sites are located in key positions at β/α-interfaces. Interestingly the sites that show significant abundance (acK392 > 10% and acK 456, 466 and 469 ≥ 1%) and are located in the C-terminal domains of α and β subunits and in close proximity to the regulatory nucleotide binding site in subunit α and the possible binding site of the extended ε subunit. Together with the observation that deacetylation effects nucleotide and ε subunit binding this PTM in particular may play an important role in allowing access to nucleotides in the regulatory binding sites.

## Discussion

In our previous study^24^ we uncovered the effect of phosphorylation on the cATPase and found that this PTM is important for stability of the intact enzyme and also affects nucleotide binding in the catalytic interface of the α/β head. Here we have extended this study uncovering additional phosphorylation sites as well as a high number of acetylation sites in all protein subunits. Acetylation is typically much less understood than phosphorylation and with up to ten acetylation sites in the α subunit, the degree of acetylation exceeds that of phosphorylation. While it has to be considered that phosphorylation sites are prone to hydrolysis during preparation and MS analysis the extent of acetylation was surprising given the fact that this modification was at first thought to be restricted to histones as part of gene regulation. However, large-scale studies of whole human and plant cell lysates have identified a high number of acetylation sites in mitochondrial and chloroplastic proteins^27^,^38^. These sites have been suggested to control photosynthesis, giving rise to the proposal that acetylation represents an important regulatory factor for photosynthetic chain complexes.

In our study, disregarding N-terminal acetylation, most of the PTMs are located in structured regions of the proteins indicating that their functional role is orientated towards stability of the complex rather than controlling the cell cycle^39^. For both acetylation and phosphorylation, we found that the stability of the complexes reduced after removal of PTMs. However, the impact of the two PTMs on complex stability differs. While phosphorylation appears to be important for global stability of the cATPase, the effect of acetylation is local, centred on the ε and δ subunits with most other protein interfaces remaining unchanged upon deacetylation. Selective loss of subunits ε and δ together with conformational changes in subunit ε associated with extended forms, were observed in the presence of deacetylase. The location of cross-links in our previous study^24^, together with high-resolution structures, locate the extended ε subunit close to the F_1_ head. It is possible therefore that the ε subunit could influence nucleotide binding by acting cooperatively with the α and β subunits to control depletion of nucleotides following deacetylation.

In a previous study chemical modification revealed residues in close proximity to the nucleotide binding site which are important for the activity of the enzyme and its ADP/ATP binding properties^40^. Interestingly we found that two of these residues (α-K266 and β-K359) are acetylated in our experiments. Deacetylation and dephosphorylation of cATPase lead to loss of nucleotides suggesting that removal of PTMs in the respective interfaces (β/α and α/β) provides access to the nucleotide binding sites and thus destabilises interactions with ATP/ADP. One of these residues (β-K359) is located in a highly modified (acetylated) region within the β/α-interface (Fig. 6) suggesting that loss of nucleotides is enhanced due to the cooperative interplay of several acetylation sites.

Our results therefore corroborate and extend previous studies. N-terminal acetylation of the ε subunit was proposed as a regulatory factor of cATPase following proteomics observations that the abundance of non-acetylated ε was reduced in drought stressed leaves^41^. This observation, together with our results, implies that N-terminal acetylation stabilises the ε subunit close to the membrane ring while deacetylation promotes unfolding/extension of the ε subunit consistent with braking mechanisms proposed earlier for this enzyme^31^,^32^,^33^,^34^. Similarly for subunit δ, a regulatory role was proposed for its homologue OSCP in directing the enzymatic activity of the bovine mitochondrial ATPase^20^. Deacetylation via SIRT3 was suggested to lead to changes in interactions between OSCP and subunit b, which may act to control the enzyme. A similar role is envisioned for subunit δ wherein interactions with subunits in the soluble head are destabilised following deacetylation.

The relevance of the phosphorylation and acetylation sites identified here is also underpinned by their conservation in *Arabidopsis thaliana*^27^. Sites that we identified and are conserved mostly cluster in specific regions of the protein sequence including the C-terminus of subunit α or the N-terminus of subunit β. Clustering of PTMs has been reported previously in other systems^42^,^43^ and suggests that modified sites may serve either as alternative sites or act synergistically to enhance their effects. Given the dramatic reduction in stability of the enzyme that was simultaneously dephosphorylated and deacetylated it seems likely that a synergistic mechanism is operative here to stabilise the enzyme through enhanced post-translational modification for survival during adverse conditions.

Interestingly, phosphorylation sites, and the most abundant of the acetylation sites, are located in catalytic (α/β) and regulatory (β/α) interfaces respectively. This concentration of different PTMs in distinct interfaces within the same complex is surprising and to our knowledge has not been described previously for cATPase or any other ATPases. Given the fact that cATPases have an additional requirement for activation/deactivation during light/dark/drought conditions, as well as the universal regulatory mechanisms needed for rotary ATPases, a more complex control system could be operative here. As in other cellular regulatory mechanisms, multiple PTMs play an important role in fine-tuning enzymes through the control of distinct phosphorylation and acetylation sites. We propose that their synergistic effects on local and global stability, as well as on catalytic and regulatory nucleotide binding sites, invoke a cross-talk between phosphorylation and acetylation sites that control rotary ATPase function under a variety of external conditions.

## Methods

### Purification of the cATPase from spinach leaves

The cATPase was purified from spinach leaves as previously described^24^,^25^.

### Gel electrophoresis

The proteins were separated by gel electrophoresis using the NuPAGE system (Invitrogen) according to the manufacturer’s protocol.

### Tryptic digestion of proteins

Proteins were digested in-gel after gel electrophoresis as described before^44^, or in-solution after ethanol precipitation using RapiGest surfactant (Waters) according to the manufacturer’s protocol.

### LC-MS/MS

Generated peptides were separated by nano-flow reversed-phase liquid chromatography (EASY nLC 1000, Thermo Scientific; mobile phase A, 0.1% (v/v) formic acid (FA)/5% (v/v) DMSO; mobile phase B, 100% (v/v) ACN/0.1% (v/v) FA/5% (v/v) DMSO) coupled to a Q Exactive Hybrid Quadrupole-Orbitrap mass spectrometer (Thermo Scientific). The peptides were loaded onto a trap column (5 mm, PepMap RSLC, C18, 300 μm I.D. particle size 3 μm; Thermo Scientific) and separated with a flow rate of 200 nL/min on an analytical C18 capillary column (50 cm, PepMap RSLC, EASY-spray column, C18, 75 μm I.D. particle size 3 μm; Thermo Scientific), with a gradient of 7–30% (v/v) mobile phase B over 30 min and a column temperature of 45 °C. Peptides were directly eluted into the mass spectrometer.

Typical mass spectrometric conditions were: spray voltage of 2.1 kV; capillary temperature of 320 °C. The LTQ-Orbitrap XL was operated in data-dependent mode. Survey full scan MS spectra were acquired in the orbitrap (m/z 350−1500) with a resolution of 70,000 an automatic gain control (AGC) target at 3 × 10^6^. The ten most intense ions were selected for HCD fragmentation in the orbitrap at an AGC target of 50,000. Singly charged ions and ions with unknown charge states were excluded from the analysis. For cross-linking doubly charged ions were included or excluded in the analysis.

### Database searching for identification of acetylation and phosphorylation sites

Raw data were searched against the SwissProt database (542,782 sequences) using the Mascot v2.4.1 search engine (Matrix Science) or against the Uniprot database (taxonomy filter *Spinacia oleracea*) using MaxQuant^36^,^45^ software v1.4.3.17.

For Mascot search the mass accuracy filter was 7 ppm for precursor ions and 0.1 Da for MS/MS fragment ions. Peptides were defined to be tryptic with maximal two missed cleavage sites. Carbamidomethylation of cysteines and oxidation of methionine residues as well as acetylation of lysine and the protein N-terminus or phosphorylation of serine, threonine and tyrosine were allowed as variable modifications.

For MaxQuant analysis the mass accuracy filter was 20 ppm for precursor ions in the orbitrap and 0.5 Da for fragement ions in the ion trap. Peptides were defined to be tryptic with a maximum of 2 missed cleavage sites. Carbamidomethylation of cysteine was set to be a fixed modification; oxidation of methionine, acetylation of lysine and the protein N-terminus and phosphorylation of serine, threonine and tyrosine were allowed as variable modifications. A decoy database search was also performed (reversed database) and an FDR of 1% was assumed.

### Mass Spectrometry of intact cATPase

Prior to MS analysis cATPase aliquots in n-dodecyl-β-D-maltopyranoside-containing buffer were exchanged against 200 mM ammonium acetate using Micro Bio-spin 6 columns (Bio Rad). Mass spectra were acquired in replicates on a Q-ToF II mass spectrometer (Waters) modified for high masses^28^ using gold-coated glass capillaries^46^. Optimized instrument parameters were as follows: capillary voltage 1.7 kV, cone voltage 190 V, extractor 5 V, source backing pressure 7–10 mbar and a collision cell pressure of 10 psi. Collision cell energy was 150–200 V. Spectra were processed using MassLynx v4.1 and spectra were analysed using Massign^47^. Representative mass spectra are shown for each experiment.

### Deacetylation of the cATPase

20 μl cATPase (approx. 10 μM) was deacetylated by addition of 1.25 μg human recombinant SIRT3 enzyme (Cambridge Bioscience). The solution was incubated at 37 °C for 2 hrs. For analysis of intact complexes, the buffer was exchanged to 200 mM ammonium acetate using Micro Bio-spin 6 columns and the complexes were analysed as described above. For identification/quantification of acetylation sites the proteins were (i) separated by SDS-PAGE followed by in-gel digestion, or (ii) precipitated with ethanol followed by in-solution digestion using RapiGest surfactant (see above).

### Chemical cross-linking

After deacetylation/dephosphorylation the cATPase was cross-linked by addition of 5 μl of 2.5 mM BS3-d0 or BS3-d4. The reaction solution was incubated at 25 °C and the aliquots processed under different conditions (i.e. after deacetylation/dephosphorylation or control) were pooled 1:1. The proteins were precipitated with ethanol and digested with RapiGest (see above). The mixture of tryptic peptides and cross-linked di-peptides was analysed by LC-MS/MS as described above. Raw data were converted into mgfs using pXtract (http://www.pfindstudio.com/software/pXtract/index.html) and cross-links were identified and quantified as described^23^. pLink search settings were as follows: instrument spectra, HCD; enzyme, trypsin; max. missed cleavage sites, 3; variable modifications, oxidation (methionine) and carbamidomethylation (cysteine); cross-linker, BS3 (light (d0) and heavy (d4)); min. peptide length, 4; max. peptide length, 100; min. peptide mass, 400 Da; max. peptide mass, 10,000 Da; FDR, 1%. Average ratios reflecting changes in protein interactions were calculated from extracted ion chromatograms of several peptide mass spectra (including different charge states).

### Simultaneous deacetylation and dephosphorylation

20 μl cATPase (approx. 10 μM) was deacetylated by addition of 1.25 μg human recombinant SIRT3 enzyme (Cambridge Bioscience). The solution was incubated at 37 °C for 1 hr. Then, 100–250 units of CIP (New England Biolabs) were added and the solution was incubated 37 °C for another hour. A control sample was processed in parallel. Instead of SIRT3 or CIP the respective buffer was added. cATPase was then cross-linked with BS3 or the buffer was exchanged for MS of the intact complexes.

### Quantification of acetylation sites

Acetylation sites were quantified using MaxQuant software v1.4.3.17 employing the described parameters (see above). For quantification of acetylation sites, a normalisation factor was calculated from unnormalised and unmodified peptide ratios of cATPase proteins. The normalisation factor was applied to unnormalised acetylated peptides.

## Additional Information

**How to cite this article:** Schmidt, C. *et al*. Acetylation and phosphorylation control both local and global stability of the chloroplast F_1_ ATP synthase. *Sci. Rep.* **7**, 44068; doi: 10.1038/srep44068 (2017).

**Publisher's note:** Springer Nature remains neutral with regard to jurisdictional claims in published maps and institutional affiliations.

## Supplementary Material

Supplementary Information
