# Supplementary material for: Acetylation and phosphorylation control both local and global stability of the chloroplast F1 ATP synthase
Source: Sci Rep. 2017 Mar 9;7:44068. doi: 10.1038/srep44068 (PMC5343439; doi:10.1038/srep44068)
Supplement: Supplementary Information [file srep44068-s1.pdf]

Supplementary Information

Acetylation and phosphorylation control both local and global stability of the chloroplast F_1 ATP synthase

Carla Schmidt[1,3*], Victoria Beilsten-Edmands[1], Shabaz Mohammed[1,2], and Carol V. Robinson[1,*]

[1] Department of Chemistry, University of Oxford, Oxford, United Kingdom.

[2] Department of Biochemistry, University of Oxford, Oxford, United Kingdom.

[3]present address: HALOmem, Martin Luther University Halle-Wittenberg, Kurt-Mothes-Str. 3, 06120 Halle, Germany.

*correspondence:

carla.schmidt@biochemtech.uni-halle.de and carol.robinson@chem.ox.ac.uk

Tables

Table S1: PTMs identified in the cATPase. The protein subunit, the modified residue, the type of PTM, the identified peptide sequence and the highest observed Mascot and MaxQuant score are given. It is indicated if the respective PTMs were identified before. Note that some modified sites could not unambiguously be assigned.

Subunit	Site	PTM	Peptide sequence	max Mascot score	max MaxQuant score	identified previously
α	N-Term	acetylation	*ATIRADEISK	57	137	
	N-Term	acetylation	*ATIRADEISKIIR	59		
	K25	acetylation	EVKVVNTGTVLQVGDGIAR	78	99	
	K114	acetylation	VINALAKPIDGRGEITASESR	19	42	
	K114	acetylation	VINALAKPIDGR	52		
	T181/183	phosphorylation	TAVATDTILNQQGQNVICVYVAIGQK	168	133	
	K266	acetylation	HTLIIYDDLSKQAQAYR	42	95	
	K374	acetylation	VGSAAQIKAMK	66	59	
	K384	acetylation	LKLELAQFAELEAFAQFASDLDKATQNQLAR		239	
	K422	acetylation	ELLKQPQSAPLTVEEQVMTIYTGTNGYLDSLELDQVR	17	63	
	K422	acetylation	ELLKQPQSAPLTVEEQVMTIYTGTNGYLDSLELDQVRK	40	68	
	Y445	phosphorylation	QPQSAPLTVEEQVMTIYTGTNGYLDSLELDQVR		40	
	K456	acetylation	KYLVELR	43	101	Schmidt et al.
	K466	acetylation	TYVKTNKPEFQEIISSTK	60	70	
	K469	acetylation	TYVKTNKPEFQEIISSTK	49	61	
	K469	acetylation	TNKPEFQEIISSTK	72	75	
	K480	acetylation	TNKPEFQEIISSTKTFTEEAEALLK	31		
β	N-Term	acetylation	*MRINPTTSDPGVSTLEK	74	125	
	N-Term	acetylation	*MRINPTTSDPGVSTLEKK	54	86	
	K17	acetylation	INPTTSDPGVSTLEKK		66	
	K18	acetylation	INPTTSDPGVSTLEKK	100		
	K50	acetylation	MPNIYNALIVK	50		

β	T54	phosphorylation	DTAGQPMNVTCEVQQLLGNNR	106	101	Schmidt et al.
	T54	phosphorylation	GRDTAGQPMNVTCEVQQLLGNNR	69	144	Schmidt et al.
	T54	phosphorylation	DTAGQPMNVTCEVQQLLGNNRVR		93	
	T62	phosphorylation	DTAGQPMNVTCEVQQLLGNNR	62	100	
	T62	phosphorylation	GRDTAGQPMNVTCEVQQLLGNNR	64		
	K145	acetylation	SAPAFTQLDTKLSIFETGIK	38		
	K217	acetylation	TREGNDLYMEMKESGVINEQNIAESK		90	
	K359	acetylation	GLAAKGIYPAVDPLDSTSTMLQPR	52		
	K392	acetylation	VKETLQR	43	85	Schmidt et al.
	K399	acetylation	YKELQDIIAILGLDELSEEDRLTVAR		215	
γ	K55	acetylation	DRIGSVK	25		
	K65	acetylation	ITEAMKLVAAAK		49	
	K71	acetylation	LVAAAKVR	36	59	
	K117	acetylation	KVALMVVTGDR	66	47	
	K150	acetylation	KLGVDYTIISIGK	87	122	Schmidt et al.
	K150	acetylation	KLGVDYTIISIGKK	88	155	Schmidt et al.
	K162/163	acetylation	KLGVDYTIISIGKK	54		
	K163	acetylation	KGNTYFIR	46	110	
	S223	phosphorylation	SDPVIHTLLPLSPK	60	66	
	S234	phosphorylation	SDPVIHTLLPLSPK	48	88	
	K335	acetylation	KTLSINYNR	70	172	
δ	S97	phosphorylation	YASALADVADVTGTLEATNSDVEK	153	228	Schmidt et al.
	K101	acetylation	YASALADVADVTGTLEATNSDVEKLIR		44	
	T175	phosphorylation	ITGTEVAVVTSVVKLENDHLAQIAK	82		
	K196	acetylation	LENDHLAQIAKGVQK	37	96	
	S216	phosphorylation	IKTVIDPSLVAGFTIRYGNEGSK	64		
ε	N-Term	acetylation	*TLNLCVLTPNR	80	170	
	N-Term	acetylation	*TLNLCVLTPNRSIWNSEVK		136	
	T2	phosphorylation	TLNLCVLTPNR	68		

ε	S85	phosphorylation	GSDIDPQEAQQTLEIAEANLR	103	82	
	K112	acetylation	QKIEANLALR	57	105	Schmidt et al.
	K112	acetylation	RQKIEANLALR	20	106	Schmidt et al.
	K112	acetylation	QKIEANLALRR	57	73	Schmidt et al.
I	K73	acetylation	GKAIEQLEK	49	89	
	K86/87	acetylation	LKKVEMDADQFR		54	
	K87	acetylation	KVEMDADQFR	82	103	Schmidt et al.
	K87	acetylation	KVEMDADQFRVNGYSEIER	79	60	Schmidt et al.
	K107	acetylation	EKMNLINSTYK	78	94	
	K125	acetylation	TLEQFENYKNETIQFEQQK	41	116	
	K135	acetylation	TLEQFENYKNETIQFEQQKAINQVR	96	94	Schmidt et al.
	T155	phosphorylation	VFQQALQGALGTLNSCLNNELHLR	73	147	
II	K124	acetylation	DASIKEQLSGVK	75	106	
	K124	acetylation	DASIKEQLSGVKDTSSEVK	63	112	
	K131	acetylation	EQLSGVKDTSSEVK	67	125	Schmidt et al.
	K164	acetylation	KETQLEVEAK		86	
	K173	acetylation	KETQLEVEAKLAEGR		63	
	K138	acetylation	DTSSEVKQLEEQANAVMR	83		
	K161	acetylation	AEISAALNKMK		52	
	K163	acetylation	AEISAALNKMK	64		
	K173	acetylation	ETQLEVEAKLAEGR	58		
	K180	acetylation	KKIEVELQEALGSLEQQKEDTIK	73		
	K180	acetylation	KIEVELQEALGSLEQQKEDTIK	40	135	
	S205	phosphorylation	SLDSQISALSDDIVKK		44	
	S205	phosphorylation	SLDSQISALSDDIVKK	70	98	
	S208	phosphorylation	SLDSQISALSDDIVKK	62		
IV	K156	acetylation	KGLGYFGK	46	130	

Table S2: Comparative cross-linking of naturally modified and deacetylated chloroplast ATP synthase. The cross-linked protein subunits, the cross-linked residues, the number of obtained spectra and the ratio of naturally modified (d0) versus deacetylated (d4) chloroplast ATP synthase are given. Changes in protein interactions > 2-fold are highlighted in grey.

Protein 1	Protein 2	Residue 1	Residue 2	# spectra	d0/d4
α	α	11	25	7	0.94
		11	114	1	0.32
		11	466	1	1.21
		25	114	4	1.76
		374	378	3	1.09
		378	384	1	0.86
		456	466	1	1.31
		456	469	1	0.56
		456	491	2	0.92
		469	491	1	0.64
α	β	11	17	1	1.71
		11	392	4	1.40
		374	426	1	1.17
		382	495	1	0.77
		456	359	3	1.04
		469	359	1	1.33
α	δ	2	101	8	0.99
α	I	2	125	6	0.67
		114	135	15	0.68
		466	116	1	0.71
α	II	456	164	1	0.59
β	β	18	50	11	1.10
		359	392	3	0.74
		359	399	4	0.52
		359	426	2	0.73
		392	399	3	0.44
		392	426	5	1.52
β	δ	18	205	3	1.09
		359	210	1	1.38
β	γ	359	55	1	1.05
		392	55	2	2.00
		426	71	2	1.20
		426	149	1	0.91
β	I	426	80	1	1.02
β	II	426	131	1	2.56
δ	δ	210	231	1	1.33
ε	ε	2	20	2	7.17
		105	112	2	204.21
ε	III	112	48	6	1.22

γ	γ	55	260	1	2.15
		55	334	2	0.76
		55	335	4	0.95
		59	334	1	0.76
		140	163	1	1.65
		140	222	1	1.26
		140	245	5	1.15
		140	263	3	1.46
		222	245	6	0.71
		245	260	1	0.94
I	I	80	87	8	1.49
I	II	73	131	12	0.79
		73	138	7	0.73
		107	161	2	1.00
		107	173	25	1.33

Table S3: Comparative cross-linking of naturally modified and deacetylated/dephosphorylated chloroplast ATP synthase. The cross-linked protein subunits, the cross-linked residues, the number of obtained spectra and the ratio of naturally modified (d0) versus deacetylated (d4) chloroplast ATP synthase are given.

Protein 1	Protein 2	Residue 1	Residue 2	# spectra	d0/d4
α	α	378	491	1	2.08
α	beta	382	495	1	4.17
α	delta	11	101	1	2.77
α	I	2	125	1	2.96
β	β	18	50	4	5.45
		359	17	1	0.25
		359	392	4	5.76
δ	δ	101	200	1	3.86
		200	210	1	1.88
γ	γ	55	335	3	1.20
		140	222	2	3.17
		140	245	1	8.23
		222	245	2	1.77
I	I	80	87	12	3.55
		87	107	1	1.00
I	II	73	131	12	5.01
		73	138	7	3.28
		107	161	1	4.27
		107	173	6	1.71

Table S4: Abundance of identified PTMs in the cATPase. The Stoichiometries of the modified sites were obtained using MaxQuant software. The protein subunit, the modified residue/site, the peptide sequence, the ratio of unmodified vs modified peptides (intensities) and the percentage of the modification as well as the average in three replicates are given. Note that some modified sites identified in this study (**Table S1**) could not be quantified.

Subunit	Site	Peptide sequence	Ratio unmodified/modified			Per cent modified			Average
			1	2	3	1	2	3	
α	*ac*K469	TN**K**PEFQEIISSTK	40.60	13.23	6.45	2.40	7.03	13.42	7.62
	*ac*K456	**K**YLVELR		19.05	1348.67		4.99	0.07	1.69
	*ac*K466	TYV**K**TNKPEFQEIISSTK	119.14	26.34		0.83	3.66		1.50
	*ac*K422	ELL**K**QPQSAPLTVEEQVMTIYTGTNGYLDSLELDQ VRK	56.55		89.90	1.74		1.10	0.95
	*ac*K384	L**K**LELAQFAELEAFAQFASDLDKATQNQLAR	42.71			2.29			0.76
	*ac*K266	HTLIIYDDLS**K**QAQAYR	116.73			0.85			0.28
	*ac*K25	EV**K**VVNTGTVLQVGDGIAR	301.50			0.33			0.11
β	*ac*K392	V**K**ETLQR	0.05			95.55			31.85
	*ac*K217	TREGNDLYMEM**K**ESGVINEQNIAESK	180.33			0.55			0.18
	*ac*K17	INPTTSDPGVSTLE**K**K		335.50			0.30		0.10
	*ac*K399	Y**K**ELQDIIAILGLDELSEEDRLTVAR			1236.28			0.08	0.03
γ	*ac*K335	**K**TLSINYNR		189.59	55.29		0.52	1.78	0.77
	*ac*K163	**K**GNTYFIR		93.91	125.73		1.05	0.79	0.61
	*ac*K150	**K**LGVDYTIISIGKK	616.52	339.97	270.73	0.16	0.29	0.37	0.27
	*ac*K71	LVAAA**K**VR	166.34			0.60			0.20
	*ac*K65	ITEAM**K**LVAAAK		220.41			0.45		0.15
	*ac*K117	**K**VALMVVTGDR		711.29	521.51		0.14	0.19	0.11
δ	*ac*K196	LENDHLAQIA**K**GVQK			8.06			11.04	3.68
	*ac*K101	YASALADVADVTGTLEATNSDVE**K**LIR			120.47			0.82	0.27
ε	*ac*K112	Q**K**IEANLALR	1540.38	31.51	244.23	0.06	3.08	0.41	1.18
I	*ac*K87	**K**VEMDADQFR		391.42	60.27		0.25	1.63	0.63
	*ac*K107	E**K**MNLINSTYK		704.23	73.15		0.14	1.35	0.50
	*ac*K86 / *ac*K87	L**KK**VEMDADQFR			434.31			0.23	0.08

I	*ac*K135	TLEQFENYKNETIQFEQQ**K**AINQVR			3460.93			0.03	0.01
II	*ac*K164	**K**ETQLEVEAK			7.06			12.40	4.13
	*ac*K161	AEISAALN**K**MK			30.62			3.16	1.05
	*ac*K135	TLEQFENY**K**NETIQFEQQK	41.95	689.04	203.88	2.33	0.14	0.49	0.99
	*ac*K173	ETQLEVEA**K**LAEGR		37.14			2.62		0.87
	*ac*K180	**K**IEVELQEALGSLEQQKEDTIK		730.67	120.73		0.14	0.82	0.32
	*ac*K124	DASI**K**EQLSGVK		543.69	618.62		0.18	0.16	0.11
	*ac*K131	EQLSGV**K**DTSSEVK		4584.63	4414.43		0.02	0.02	0.01
IV	*ac*K156	**K**GLGYFGK		1630.18	595.45		0.06	0.17	0.08
α	*p*T181	TAVA**T**DTILNQQGQNVICVYVAIGQK		0.66			1.54		0.51
	*p*T183	TAVATD**T**ILNQQGQNVICVYVAIGQK	0.73			1.37			0.46
β	*p*T62	GRDTAGQPMNV**T**CEVQQLLGNNR		134.58	25.20		0.007	0.040	0.02
	*p*T54	D**T**AGQPMNVTCEVQQLLGNNR			1245.35			0.0008	0.0003
γ	*p*S234	SDPVIHTLLPL**S**PK		9324.88	6716.37		0.0001	0.0001	0.0001
δ	*p*S97	YASALADVADVTGTLEATN**S**DVEK		592.45			0.002		0.001
I	*p*T155	VFQQALQGALG**T**LNSCLNNELHLR	0.40	698.03	8.10	2.51	0.001	0.12	0.88
II	*p*S205	SLD**S**QISALSDDIVKK	4398.89	15209.36		0.0002	0.00007		0.0001
	*p*S208	SLDSQI**S**ALSDDIVKK		43630.02			0.00002		0.00001

Figures

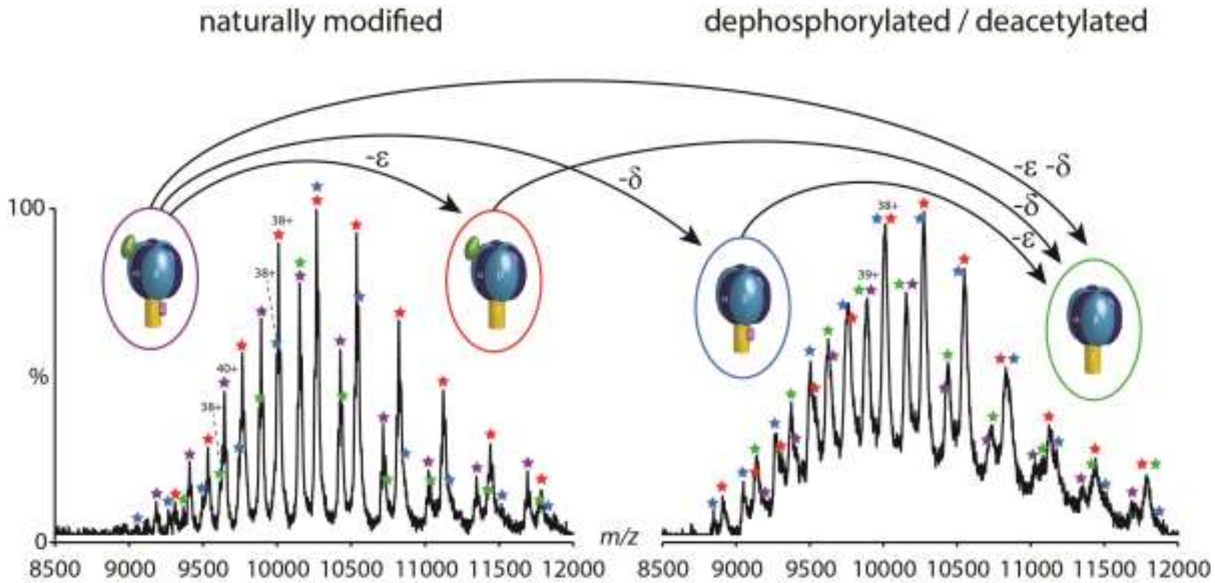

Figure S1: Simultaneous dehosphorylation and deacetylation. Spectra of the naturally modified **(lhs)** and the dephosphorylated/deacetylated **(rhs)** cATPase are shown. Four subcomplexes of cATPase were identified.

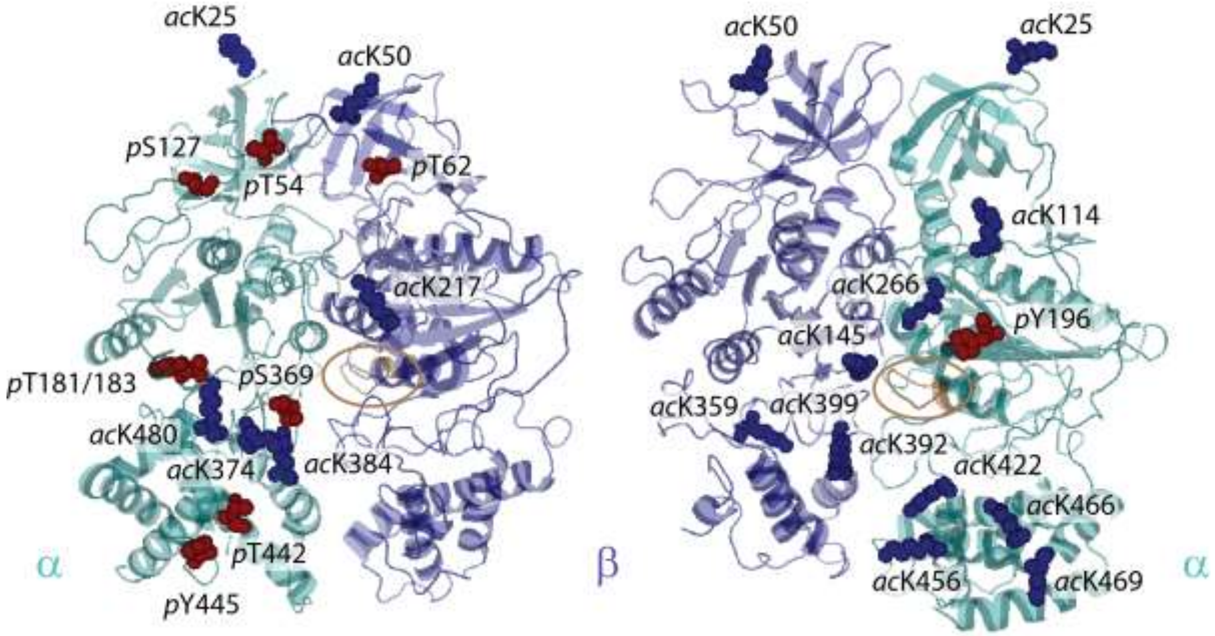

Figure S2: Phosphorylation and acetylation sites are located at the protein interfaces. The α/β and β/α interfaces of the cATPase are shown (PDB ID XY). Phosphorylated (red) and acetylated (blue) residues are shown (space fillings). Most phosphorylation sites are located in the α/β interface, while acetylation is more prevalent in the β/α interface.
